# Analysis of human immune responses in quasi-experimental settings: tutorial in biostatistics

**DOI:** 10.1186/1471-2288-12-1

**Published:** 2012-01-03

**Authors:** Rajiv Sarkar, Sitara S Ajjampur, Honorine D Ward, Gagandeep Kang, Elena N Naumova

**Affiliations:** 1 Christian Medical College, Vellore, India; 2 Tufts Medical Center, Boston, MA, USA; 3 Tufts University School of Engineering, Medford, MA, USA

## Abstract

**Background:**

Human immunology is a growing field of research in which experimental, clinical, and analytical methods of many life science disciplines are utilized. Classic epidemiological study designs, including observational longitudinal birth cohort studies, offer strong potential for gaining new knowledge and insights into immune response to pathogens in humans. However, rigorous discussion of methodological issues related to designs and statistical analysis that are appropriate for longitudinal studies is lacking.

**Methods:**

In this communication we address key questions of quality and validity of traditional and recently developed statistical tools applied to measures of immune responses. For this purpose we use data on humoral immune response (IR) associated with the first cryptosporidial diarrhea in a birth cohort of children residing in an urban slum in south India. The main objective is to detect the difference and derive inferences for a change in IR measured at two time points, before (pre) and after (post) an event of interest. We illustrate the use and interpretation of analytical and data visualization techniques including generalized linear and additive models, data-driven smoothing, and combinations of box-, scatter-, and needle-plots.

**Results:**

We provide step-by-step instructions for conducting a thorough and relatively simple analytical investigation, describe the challenges and pitfalls, and offer practical solutions for comprehensive examination of data. We illustrate how the assumption of time irrelevance can be handled in a study with a pre-post design. We demonstrate how one can study the dynamics of IR in humans by considering the timing of response following an event of interest and seasonal fluctuation of exposure by proper alignment of time of measurements. This alignment of calendar time of measurements and a child's age at the event of interest allows us to explore interactions between IR, seasonal exposures and age at first infection.

**Conclusions:**

The use of traditional statistical techniques to analyze immunological data derived from observational human studies can result in loss of important information. Detailed analysis using well-tailored techniques allows the depiction of new features of immune response to a pathogen in longitudinal studies in humans. The proposed staged approach has prominent implications for future study designs and analyses.

## Background

Human immunology is a growing field and includes methodologies of many experimental and clinical disciplines: molecular biology, microbiology, immunogenetics, clinical immunology, pathophysiology, epidemiology, and potentially others. The essence of scientific proof in human immunology employs a set of applicable and ethically acceptable rules. The direct interpolation of techniques developed for fully controlled experimental designs can be a challenging task. The profound differences in clinical, epidemiological, and laboratory studies have to do with basic assumptions, which logically define a research hypothesis and analytical procedures we apply to test this hypothesis. For example, in a study aimed to examine the effect of "an event", say "infection by a pathogen" on a marker of an immune response such as antibody levels, a design or protocol for measuring such an effect in a fully controlled experiment may differ dramatically in a murine model and in a cohort of newborn children. It is important to know if the measurements that are used to judge the effect were obtained from the same subjects or not, because this aspect of a study design will affect the choice of the statistical test. If a study subject contributes two measurements: one before "an event of interest", also called a baseline measure and another measurement taken after an event, we are dealing with a so-called "repeated measurement" scenario, a pre-post design, which is the focus of this communication.

Reviewing the recent literature one can easily notice that a typical statistical analysis conducted in a traditional pre-post design is often limited to a paired *t*-test. In some instances this analytical scheme is sufficient. However, in many situations it can be overly simplistic since it does not take into account the complexity of data collection protocols and various issues related to field research, ignores underlying theoretical assumptions that are essential for proper use of statistical tests, and discounts the important confounding factors associated with immune responses in humans. In this communication we address key questions of quality and validity of a statistical analysis performed by measuring human immune responses in a longitudinal setting. By using an example, we provide step-by-step instructions, and describe the pitfalls and solutions in a comprehensive examination of data.

The organization of the manuscript is as follows. First, we will introduce an example borrowed from an actual study to detail the study design, experimental procedures, and notations. Next, we present the traditional statistical approach to a pre-post comparison of study outcomes in controlled experimental conditions, and describe our analytical plan in several steps with an emphasis on developing supportive visual aids. We then present the rationale for further explorations into the nature of pre-post changes and suggest an approach to such examinations by investigating the effect of an "outlier" as an example. We detail the potential flaws of the traditional analytic approach applied to longitudinal settings of measuring human immune responses in "real-world" conditions. We focus on the important consideration of the measurements' timing, age of the subject, and seasonal effects. We demonstrate how novel analytical techniques can be applied to reveal unique features of immune response dynamics, age-specific elevation in the immune response, and the effect of seasonal synchronization that explains a large fraction of variability of temporal changes. We conclude this tutorial with recommendations for the use of this proposed scheme in other practical settings.

## Methods

### Motivating Example

To illustrate the analytical process we used data collected from a birth cohort of children observed over three years in a semi-urban community in south India. The aim of the study was to examine the change in serum IgG levels measured by ELISA units to the immunodominant gp15 antigen as a consequence of the first episode of symptomatic cryptosporidial infection [1]. A total of 452 children were recruited over an 18-month period starting in March 2002; 373 children completed the 3-year follow-up. Field-workers visited each child twice-a-week to record any morbidity. Surveillance stool samples were collected every two weeks and diarrheal stool samples were collected with each episode of diarrhea [2]. The diarrheal stool samples were examined for the presence of *Cryptosporidium *spp. by microscopy and the positive samples were subjected to PCR-RFLP for genetic characterization [3]. Fifty-three children in this cohort experienced a total of 58 episodes of confirmed cryptosporidial diarrhea, out of which 47 episodes were due to *C. hominis *(see details elsewhere [3]). For illustrative purposes, we used data from 40 children whose first episode of cryptosporidial diarrhea was due to *C. hominis *infection. For these 40 children we utlilized the results of ELISA testing in two surveillance stool samples collected *before *and *after *the child's first episode of cryptosporidial diarrhea. The original data are provided in supplemental material, which include information on IR values, sampling date and child's age (see Additional file 1). The details on measuring serum IgG levels to the gp15 antigen and normalization of ELISA units can be found elsewhere [1,4]. For the purpose of this study, serum IgG levels are used as a measure of immune response.

The main objective for the performed statistical analysis is to derive inferences from a change in the immune responses measured in ELISA units at those two time points. In statistical terms we aim to detect the difference in the markers of immune responses in a study with a pre-post design delivering two repeated measurements for each subject.

In this tutorial we use the following notations: *Y_i _*- values for immune responses for *i*- child; each *Y_i _*consists of two values: *Y*_*t*1 _- first measurement and *Y*_*t*2 _- second measurement, where *t*_1 _- time of first measurement; *t*_2 _- time of second measurement. A degree of change on an individual level is defined in three ways: as an absolute difference, Δ*Y_i _*= Y_*t*2 _- Y_*t*1_, an absolute difference of log-transformed values, Δ*Y_i _*= lnY_*t*2 _- lnY_*t*1 _and log-fold change, Δ*Y_i _*= ln(Y_*t*2 _/Y_*t*1_). We also specify t*_E _*as the time of the event of interest. Additional relevant information to the presented illustration includes age at measurement and date of measurements. Sections below demonstrate the importance of this information in better understanding the variability in immune responses.

## Results and Discussion

### Traditional Statistical Approach

A traditional statistical approach to a pre-post comparison in a controlled experiment consists of four steps. In Step 1, we examine the distributions of measures at both time points: pre- and post-event, and provide summary statistics, e.g. mean, median, standard deviation, and inter-quartile range separately for pre- and post-event measurements. In Step 2, we test for potential outliers, transform original values if necessary, and impute missing values if appropriate. In Step 3, we estimate the change for each subject, and again, provide summary statistics for the difference between pre- and post-event measurements. In Step 4, we test the significance of change using parametric or non-parametric tests.

**Figure 1:**
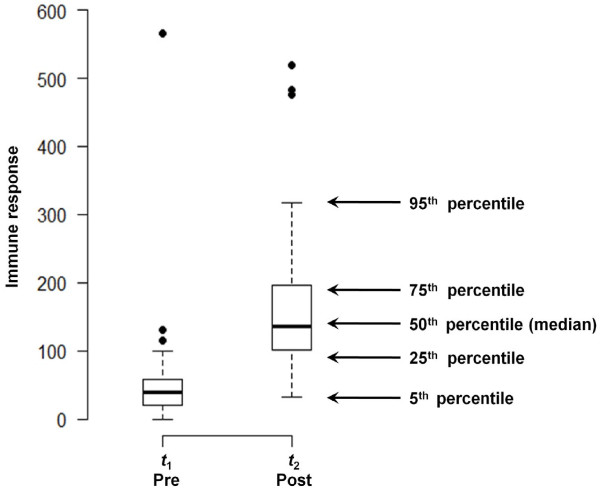
**Distribution of pre- and post- event measurements of immune responses**. The distributions are depicted by a compact box-plot indicating five summary statistics: 5^th^, 25^th^, 50^th ^(median), 75^th^, and 95^th ^percentiles, as well as potential outliers that are substantially exceeding interquartile range (IQR) (higher then 1.5*IQR). Summary statistics for pre- and post- event measurements are shown in the Table 1.

Typically, the results of the analysis are summarized in a tabular form, similar to Table 1. Simple visualization tools can greatly enhance the understanding of results and help with interpretation. For example, Steps 1 and 3 of the analysis can be easily represented by using, for example, a dot-plot, box-plot, line-plot, histogram or scatterplot. The selection of a visual display for depicting a distribution of measured outcomes mainly depends on the sample size and on the intended purposes. In modern publications, for various reasons (primarily the commonly used software packages), a dot-plot is the representation of choice. However, a distribution of measured outcomes for a sample of moderate size can be clearly presented by a compact box-plot. We provided two box-plots for our sample in Figure 1. Box-plots clearly depict five summary statistics: 5^th^, 25^th^, 50^th ^(median), 75^th^, and 95^th ^percentiles for both pre- and post- samples. The box-plots illustrate the first important observation: the distribution at a baseline (or at time *t*_1_) is more compact and has a lower median value than the distribution for the second time point (*t*_2_). Both distributions have a tendency to be skewed toward high EU values with a few distinct outliers, suggesting the potential need for some form of transformation, for example a log-transformation, which we employed in this tutorial.

**Table 1:** Summary statistics for the pre & post event immune response (IR)

	Untransformed		Natural log transformed	
	Pre	Post	Pre	Post
Mean	54.9	171.2	3.56	4.95

Standard deviation	87.9	117.1	0.86	0.65

95^th ^percentile	122.5	478.9	4.81	6.17

75^th ^percentile	57.9	196.1	4.06	5.28

50^th ^percentile	38.6	135.9	3.65	4.92

25^th ^percentile	20.1	101.9	2.99	4.63

5^th ^percentile	8.86	36.6	2.14	3.63

Interquartile range	37.8	94.1	1.06	0.65

Skewness	5.09	1.53	0.48	-0.15

Kurtosis	30.1	4.97	4.29	3.02

**Figure 2:**
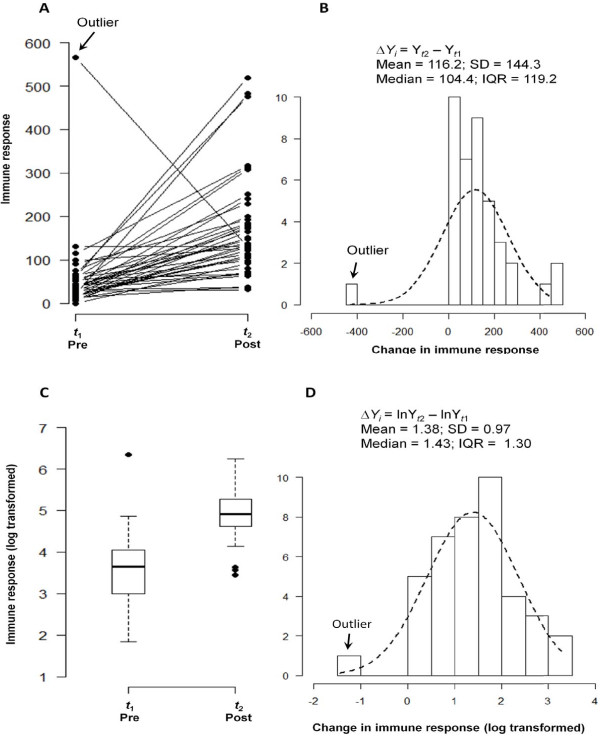
**Line graph of individual trajectories and a distribution of differences**. Individual trajectories of change in immune response measurements (Panel A) reflect a general pattern and the degree of similarity among the responses. A histogram of the observed absolute differences in pre-post measurements (Panel B) indicate systematic increase in post measurements, except one case of a negative change, marked as an outlier. The dashed line indicates approximation by a normal distribution. Summary statistics for absolute difference values are shown. The distributions of pre- and post-event measurements (as box plots) and their difference (as histogram) for log-transformed values are shown in Panel C and D respectively.

While compact and easy to interpret, box-plots do not reflect the individual changes. To correct for this, the box-plot can be supplemented with a line graph (Figure 2A), which illustrates individual trajectories and the degree of similarity among the responses. If the majority of subjects have higher (or lower) outcomes at the second time point compared to the first, we observe the overall similarity or "synchronization" in the responses. Typically, if the change is chaotic, e.g. little synchronization in the change is observed, and both distributions substantially overlap, then the conclusion of an unremarkable change is supported by the *t*-test results. However, if the distributions of outcomes for pre- and post- samples are compact and spread far apart, meaning a pronounced synchronization in the overall change is observed, then the comparison of the individual values at two time points produces a low p-value in the paired *t*-test.

A histogram of the observed absolute differences, Δ*Y_i _*= Y_*t*2 _- Y_*t*1 _(Figure 2B), describes the pattern of change. The histogram provides a clear depiction of the predominant direction of change: the majority of children exhibited an increase in IR values (except for one case with a negative value); the difference between post- and pre- values ranges between (-435.0 and 459.4), 50% of children exhibited a change of 104.4 units; 25% of children exhibited a change less than 42.4, 75% of children had a change less than 161.6, yielding an IQR of 119.2 units of IR. On average, there was an IR increase of 116.2 ± 114.3. Testing if the average change differs from zero, yields a *t*-value of 5.095 that corresponds to a p-value of < 0.001.

However, the observed skew in the distribution warrants the use of appropriate transformation and non-parametric tests that alleviate the effect of skew toward high IR values and potential outliers. In our case, a non-parametric Wilcoxon signed-rank test also yields a very low p-value of < 0.001. Next, we transform the IR data using natural log and produce box-plots for pre-event and post-event IR data (Figure 2C). The summary statistics for transformed data are presented in Table 1. The transformed data has less skew as indicated by the box-plot and the coefficients of skewness and kurtosis in Table 1. The superimposed distribution line (in Figure 2D) offers an insight on how close the observed differences are to the normal distribution. On average, the absolute difference, D*Y_i _*= lnY_*t*2 _- lnY_*t*1_, was 1.38 ± 0.97. Testing if the average change differs from zero, yields a *t*-value of 9.037 that corresponds to a p-value of < 0.001. A better way to present the expected difference is to express it as a percent increase together with the 95%confidence interval: so, in our example the average change is 38% and 95% CI is [7.5%; 69.5%].

This standard analysis provides a first glance at the nature of change in the outcome of interest. The succinct form of visuals and tables offers a comprehensive overview of results that are clearly presented to support the study conclusion of a significant increase in IR following a diarrheal episode. In this analysis we assume that the mean and variance of the change are reasonably constant throughout the range of pre-event values. We also assume that neither skew nor outliers affect the estimate of the change and the relationship between pre-and post- event values. However, to reach meaningful conclusions these assumptions should be further explored.

### Further Explorations

There is more to the behavior of individual differences that deserves further attention. In general, we assume that high post-event values simply reflect the magnitude of the immune response in that particular child. However, it is plausible that the degree of change in the immune response might depend on the baseline value. We may suspect that children with high pre-event values tend to have high post-event IR values, but the relative increment would be small. Detection of such a relationship is an important finding because it highlights the heterogeneity in the observed change.

**Figure 3:**
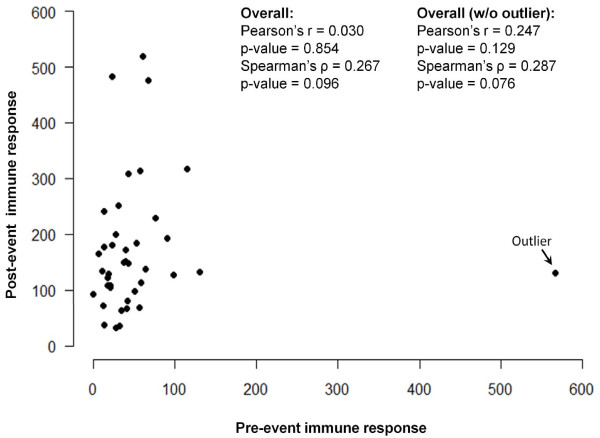
**Scatter-plot of pair measurements: the baseline IR values are shown on a horizontal axis -- labeled as "Pre-event immune response" -- and the measurements for the second time point -- labeled as "Post-event immune response" are on a vertical axis**. An outlier is marked. Values of Pearson's and Spearman's correlation coefficients are shown for all subjects with and without the outlier.

To explore the dependency of the change in immune response on the baseline values of IR, we use two scatter-plots. We plot the baseline IR values on the horizontal axis -- labeled as "Pre-event immune response" -- and the measurements for the second time point -- labeled as "Post-event immune response" -- on the vertical axis. Figure 3 shows a scatter-plot of pre- and post- values. Next, we examine the correlation between two sets of measurements using Pearson and Spearman correlation coefficients. The Spearman coefficient is based on the order, *not *the absolute values, and therefore is less sensitive to unusually high IR measurements. In the presence of heavy skew and potential outliers, these coefficients are not expected to be identical. Both correlation coefficients are low (Pearson's r = 0.030, p-value = 0.854; Spearman's ρ = 0.267, p-value = 0.096), indicating no dependency of the post-event IR on IR at baseline.

**Figure 4:**
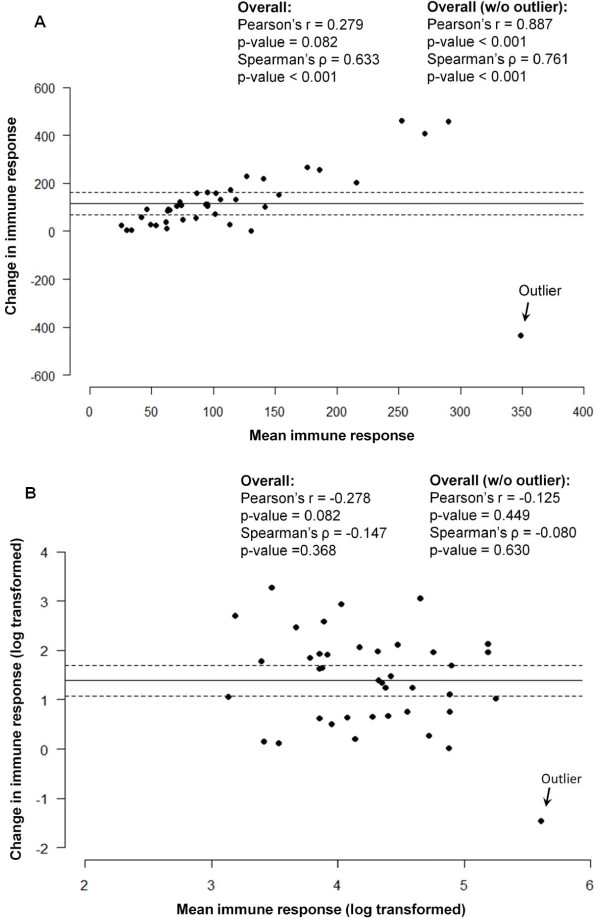
**Scatter-plot of pair measurements: Figure 4A depicts the average of pre- and post-event IR values are shown on a horizontal axis and the values for absolute difference are on a vertical axis**. The horizontal line in the middle is the mean difference between the pre- and post-event IR values, and the dotted lines are the 95% CI for the pre-post difference. Figure 4B uses the log-transformed IR values and reveals a lesser dependency. An outlier is marked. Values of Pearson's and Spearman's correlation coefficients are shown for all subjects and by subgroups with and without the outlier.

For the mean and SD of the change to be meaningful estimate we must assume that they are reasonably constant throughout the range of pre-event values. As in Bland and Altman [5] argument, the usual plot of the pre-event and the post-event IR values is inefficient, because children with high pre-event values might have high post-event IR values, but the relative increment can be small or vice versa, i.e. children with high increment will express very high post-event values. Figure 4A demonstrate the relationship between the difference and average for the actual values of IR, whereas Figure 4B uses the log-transformed IR values and reveals a lesser dependency. This suggests that the change in actual values linearly increases with an increased IR and is scale-sensitive. However, we need to be careful when interpreting the changes in log-transformed IR values as small absolute differences in IR can result in large differences in logged values, especially if the pre-event values are very small.

Cases with atypical behavior are often considered to be outliers and deserve special treatment. It is possible that their effect on the overall results is disproportionally strong and might bias our conclusions. Such bias has to be carefully examined. For example, Figure 2 shows that the highest EU value at baseline is associated with the largest decline in EU values: one child had an unusually high IR value before the event of interest, which later declined to approximately average IR values at the second time point. We consider this point to be an outlier based on the fact that it is biologically implausible to have a decrease in immune response following an event, unless the child had a prior exposure to the pathogen that has gone undetected. This outlier is marked in Figure 2. Now we illustrate how the effect of an outlier on the relationships between change and baseline values can be examined.

Let us flag and remove the suspicious value first noted when we plotted the baseline values in Figure 2 (marked as "outlier"). We intentionally did not emphasize the outliers for post measures in Figure 1 because the log transformation eliminates their effect [6]. Then, we recalculate the correlation between pre- and post-event values in the sample without the case exhibiting unusual behavior (Figure 3). Once again, the overall correlation is weak and non-significant, although the estimates are getting closer (Pearson's r = 0.247, p-value = 0.129; Spearman's ρ = 0.287, p-value = 0.076). This observation supports the earlier statements that the pre- and post-event IR's are not correlated.

For Figure 4A, the difference in correlations for actual values (Pearson's r = 0.279, p-value = 0.816; Spearman's ρ = 0.633, p-value < 0.001) is likely to be driven by the outlier. After removing the outlier the correlation coefficients become very high and similar (Pearson's r = 0.887, p-value < 0.001; Spearman's ρ = 0.761, p-value < 0.001). For the log-transformed data (Figure 4B) the correlations are low for a complete data set (Pearson's r = -0.278, p-value = 0.082; Spearman's ρ = 0.147, p-value = 0.366) as well as for the data with an outlier removed (Pearson's r = -0.125, p-value = 0.449; Spearman's ρ = -0.080, p-value = 0.630). This confirms the early statement that the change in actual values depends on IR and is scale-sensitive.

These findings suggest that the immune response might exhibit more complex behavior than we originally postulated. We can suspect that the study group is heterogeneous and it is quite likely that some important factors, or interactions, must be considered in order to better understand the nature of the observed relationship. The child's time of measurement relative to time of exposure and infection, and the infecting dose are potential candidates for such factors.

In this section, we illustrated how one can test the hypothesis that children with high post-event values tend to have high pre-event IR values. We also highlighted the importance of better understanding these associations in order to reach valid conclusions. In planning the pre-post experiments in human cohorts, it is essential to collect information on parameters that can identify potential interactions and/or effect modification that might affect the degree of change. There are also a number of techniques that can further improve a statistical analysis protocol, including statistical tests for the difference in correlation coefficients.

### Flaws of a Traditional Scheme: Assumption of Time Irrelevance

The traditional scheme, although simple and familiar, might be inadequate for "real-life" settings in measuring human immune response. The major flaws are in the basic assumptions that might be valid in experimental conditions but not in "field" studies. Typically, in experimental settings or in clinical settings, the time of measurement, the dose of exposure, and the timing of exposure are well controlled. In "field" or observational studies, the time of measurement, even in a well-run cohort, is subject to fluctuations, which have to be carefully considered in the analysis. Moreover, the "time of event" - in our case the time of exposure to infection with a specific pathogen - could not be predicted (unless performed in volunteer studies). Such features do not make human studies any less precise or valuable, but they do require serious attention to details and the proper selection of statistical techniques.

One of the major problems of traditional analysis is the assumption of time irrelevance. Figure 2 adequately summarizes the change in individual measures. However it implies that the measurements were taken at the same time for all pre- or post- event samples. It also may imply that the time of measurements is irrelevant for the purpose of an analysis. The use of an absolute difference of pre- and post-event measures, as a characteristic of change, implies that the observed change is solely due to the event of interest. The duration between pre- and post-event measures, even if specified, is often assumed to be irrelevant to the observed magnitude of change. In this section, we illustrate that the time of measurement is not a trivial matter and must be specifically considered.

**Figure 5:**
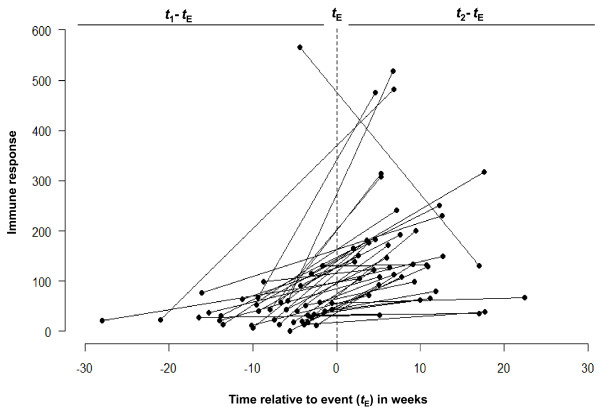
**Line graph of individual trajectories centered on the time of event reflects time elapsed between pre/post measurements**. The measurements collected before an event are on left relative to a vertical dotted line and the post-event measurements are on right side. Thus, the negative values on a horizontal axis indicate the time of a surveillance sample taken before diarrheal episode and the positive values are times of surveillance sample since an episode. The summary statistics for measurement timing and shown in Table 2.

Let us re-plot Figure 2A to capture the time of measurements taken prior to the event and post event with respect to the time of event itself. Instead of anchoring the time of pre- and post-event measurements to two arbitrary points on horizontal axes (as in Figure 2A), we plot the values of pre- and post-event measurements with respect to time of episode for each child. To do this, we convert the chronological dates of diarrhea onset and collection of blood samples for serum IgG levels into time in weeks *before *and *after *an episode. In Figure 5, all 40 pairs are aligned with respect to episode timing, so that the measurements *before *an episode will be on one side relative to a vertical dotted line, and all the measurements *after *an episode will be on other side. Thus, the negative values for "time" represent time of a sample taken before an event and the positive values represent time of a sample taken after an event. On the horizontal axis, each line starts with a negative value for time and ends with a positive value for time. The vertical axis reflects the IR values for immune response at baseline and post event. Now, we examine the average time of sample collection. On average, samples were taken 7.7 ± 5.9 weeks prior a diarrheal episode and 8.3 ± 4.9 weeks after an episode (see Table 2). No tendency for a shift toward longer or shorter time interval post event was observed (p-value = 0.660).

**Table 2:** Summary statistics for the timing of pre & post event serum sample collection (IR)

	Pre event	Post event
Mean (SD)	7.7	8.3

Standard deviation	5.9	4.9

95^th ^percentile	18.7	17.6

75^th ^percentile	10.1	11

50^th ^percentile	5.9	6.9

25^th ^percentile	3.4	4.8

5^th ^percentile	1	2.4

Interquartile range	6.6	6.2

In observational studies the consideration of timing can be quite important for reaching valid conclusions. In the simplest cases and when the mentioning of duration between pre- and post- measurements contributes to a better understanding of a process, the change in IR can be easily adjusted. This can be done by presenting the average change per unit of time, e.g. days, weeks, or months. In our example, the summary statistics for IR increase, presented in Figure 2B, will change from its absolute value of 116.2 ± 114.3 to 8.3 ± 9.0, reflecting a weekly rate of change. This is a more accurate representation of overall change in IR, and is suitable if the assumption that the rate is constant over time for each child is correct. However, it is plausible that the magnitude of IR depends on when the sample was taken after an infection, or in other words, depends on the time elapsed since the time of exposure to a pathogen or of onset of symptoms. To explore this phenomenon, we shall perform a proper analysis.

### Detection of Important Features in Immune Response Dynamics

It is well known that, in general, immune response to an antigen might exhibit a detectable increase after an antigenic challenge, reach its maximum and then decline to a baseline level or continue to be slightly elevated for some time. Such behavior is characteristic for non-linear dynamics and requires caution if traditional statistical techniques are attempted.

**Figure 6:**
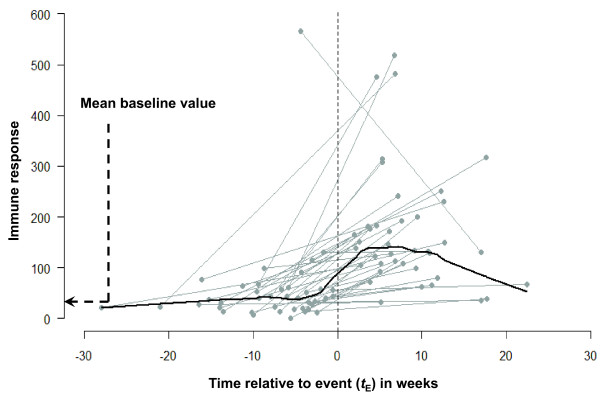
**Line graph of individual trajectories centered on the time of event with the superimposed smoothed curve illustrates the general pattern temporal change in immune responses with respect to timing of measurements**. A non-parametric (LOWESS) smoother with the window covering 1/3rd of data points is shown.

In Figure 5, the IR values for each pre-post pair are centered according to the timing of an episode. Thus, negative values on a horizontal axis indicate the time of a sample taken before a diarrheal episode and positive values are given to times of samples following an episode. This graph covers the whole range of times at which the measurements were taken and demonstrates that although the measurements taken after an episode were overall higher than the measurements taken before an episode, the degree of increase could be a complex function of time elapsed since a diarrheal episode. In order to reveal this non-linear feature we smoothed the data using statistical tools that help to describe the relationship between IR values and timing relative to diarrheal episode. For this step, we superimposed a plot with individual lines for each pair with a curve built from a model applied to small localized subsets of the data, so-called loess [7] a locally weighed least squared regression (Figure 6). The non-parametric smoother allows estimation of an average value for IR in sequences of small subsets, each covering a portion of data points. The smoothed curve reveals that the measurements obtained before the diarrheal episodes remain stable across the whole time period: from 30 weeks until at least 5 weeks prior to the diarrheal episode. Right before the episode, we start to observe a steady increase in IR responses until approximately 9 weeks after an event, when the increase reaches its maximum. Then, the curve starts to decline to the baseline level at about 20 weeks after the event. Similar results were shown in serological analysis of a cryptosporidial epidemic where the intensity of response was higher among specimens drawn 8 weeks after the first case report compared to those before or after that period [8].

This analysis helps us to define a curve without forcing a specific shape. If the episode timing is accurate and unbiased, one can model this process accounting for a discrete step with a curve consisting of two segments: one for a pre-event period and another for a post-episode period, so a step will be allowed at the event. In our case, the timing of an episode is self-reported and might contain some delay, which is potentially reflected by a rise prior to the episode.

**Figure 7:**
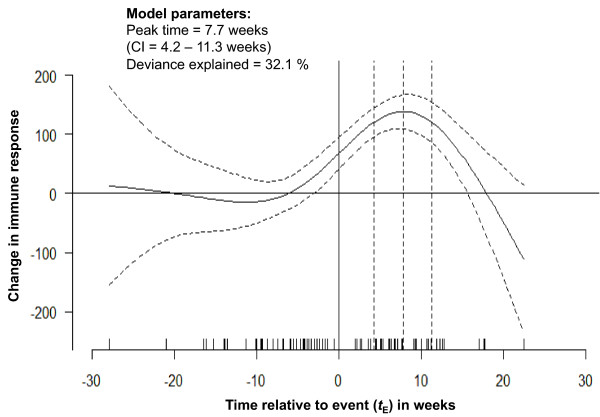
**Predicted change in immune response, based on generalized additive modeling -- with cubic-splines supported by 5 knots -- reflects the average change for the whole range of measurement timing along with the 95% confidence interval indicating the degree of uncertainty in the detected pattern**. The estimates of peak timing are shown.

To provide estimates of the curve's features and measures of the modeling uncertainty, we fit a generalized additive model [9,10] with a form similar to what we found in the previous step (with the cubic-splines supported by 5 knots). Figure 7 shows the values of IR change predicted by the model (shown with the solid black line) and the curve's confidence interval (CI) at 95^th ^percentiles (the upper and lower boundaries are shown with black dotted lines). The model explained 32% of variability in the data. The average IR value was estimated to be 58.1 units. The estimated time to peak was 7.7 weeks post-diarrhea when the IR reached its maximum of 138.1 with a standard error of 18.2. To confirm the quality of estimates, we simulated the curve for the period of 4 - 20 weeks post-diarrhea, with a refined time increment of 0.01 weeks and obtained the estimates of peak timing and found its CI to be practically identical to the presented estimates.

This analysis helps to identify important features of immune response dynamics: general stability of immune response before an episode and a non-linear dynamic of response over time with a pronounced peak followed by a decline to a baseline level. These findings have important implications. For example, they might help to optimize future study designs by better targeting the time period that captures the best immune response.

### Age as a Factor of Immune Response

Immune response can vary with age. The standard analysis can not explicitly account for potential differences in age. Even in the most closely observed study cohorts, the age at which a child contracts an infection cannot be controlled. To better understand how chronological age affects the immune response, we have to perform an analysis with an intention to detect the association between IR and the age at measurements.

**Figure 8:**
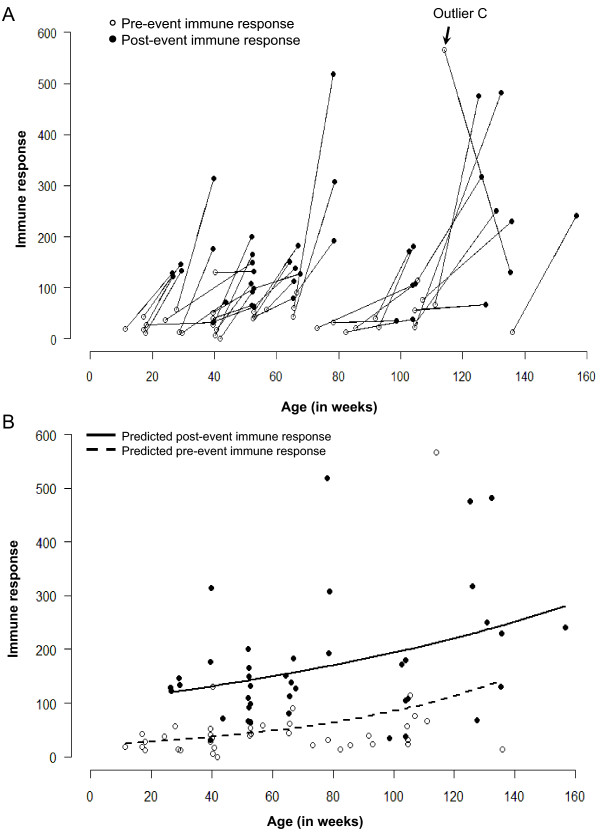
**Graph showing the immune responses of children aligned according to age. **Panel A shows the line graph of individual trajectories of immune responses. Panel B shows the predicted values for fitted lines for measurements taken before child's diarrheal episode (dashed line) and for measurements taken after an episode (solid line) -- as obtained from a log-linear regression model.

Just as we aligned the time of measurements relative to the time of an event, we can similarly align measurements to the age of a child and explore how the magnitude of response is associated with the child's age. Figure 8A shows the individual IR values in pre-post pairs for each child according to the age of measurement, reflected on the horizontal axis. For example, the youngest child in this study was 11 weeks old when his/her first measurement was taken; 15 weeks later the second measurement was taken when this child was 26 weeks old. Using another example, a child whom we consider to be an "outlier" (marked on Figures 2, and 3) was 114 and 135 weeks old at the time of the first and second measurements, respectively.

It is evident that all children except one have relatively lower IR values in the pre-samples as compared to the post-samples. Younger children tend to have a relatively smaller increase in IR than the older children. This phenomenon can be thoroughly explored. Let us re-plot Figure 8A but without the lines connecting each pair. Thus, Figure 8B shows IR values for the first measurements (empty circles) and the second measurements (solid circles) for each child. Now, we fit the best curve to each set of points separately: one curve for all measurements taken before child's diarrheal episode (dashed line) and one curve for all measurements taken after an episode (solid line). These curves represent predicted values of IR as a function of age, and indicate that IR measured before an episode (as well as after) increases as children are getting older. These predicted values were obtained from a log-linear regression model, in which an increase in variability that occurs along with an increase in IR is accounted via the Poisson assumption for error structure [11] as follows:

$$\text{log}\left(\text{E}\left[Y_{t1}\right]\right)=\beta_{0}+\beta_{1}*\text{ag}{\text{e}}_{t1}$$

Using regression coefficients as applied to pre- and post- measurements we are able to estimate the overall magnitude of an increase in IR and the increase in IR associated with one unit of age. Regardless of age after an episode of diarrhea, the IR is 4.2 times higher compared to the IR before an episode. Both pre-event and post-event IR values depend on the child's age: on average, in one year (50 weeks) IR increases by 67.8% (95% CI: 62.3%, 73.2%) in baseline measurement and by 22.3% (95% CI: 19.2%, 25.5%) in post-episode measurements. We then confirm these results by removing an outlier and also by using a generalized linear model adapted for the log-transformed values and a Gaussian error structure (data not shown).

This analysis shows strong relationships associated with age at first encounter with an infectious agent, helps to determine how age contributes to overall increase in IR, and identifies critical features in IR related to time of measurements.

### Environmental Exposure as a Factor of Immune Response

Diarrheal infections are known for their seasonal behaviors: some peak in hot summer months, some peak in cold dry winter months [12-14]. Environmental factors with seasonal effects drive the probability of exposure to pathogens, affect concentration and pathogenicity, modify susceptibility to infections; and have to be considered whenever it is possible [15]. It is rare that, in a large scale population study, such as a birth cohort, or even in a well run clinical investigation, the enrollment of study subjects can be done instantaneously or during a very short time interval. This means that during the study, subjects can be exposed to different environmental conditions, which can affect the time of an episode and modify the IR values obtained before and after an event of interest.

**Figure 9:**
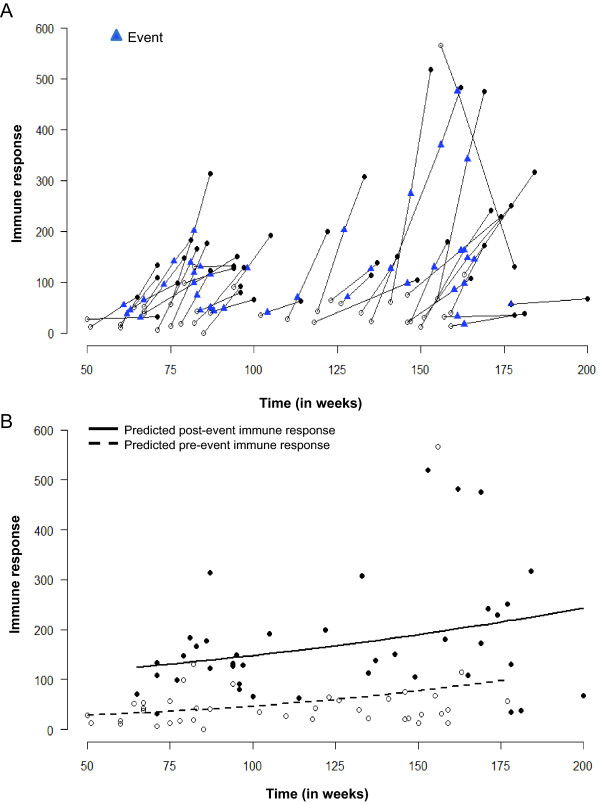
**Graph showing the immune responses of children aligned according to calendar time of measurement. ** Panel A shows the line graph of individual trajectories of immune responses. Panel B shows the predicted values for fitted lines for measurements taken before child's diarrheal episode (dashed line) and for measurements taken after an episode (solid line) -- as obtained from a log-linear regression model.

In our example, the birth cohort was recruited over 18 months and the samples were collected from March of 2002 until August of 2006 [16,17]. To detect the seasonal change in IR, we re-plotted Figure 7 with the calendar time instead of age. Figure 9A shows individual pairs with the time of measurements for pre-event sample (empty dot), post-event measurements (solid dot) and the time of diarrheal episode (blue triangle) for each child. The first measurement in this study was taken at week 50 counting from the time of the first enrollment. We perform this analysis in a manner similar to the study of association with age. When we plot the predicted curve for pre- and post- event measurements over time (Figure 9B), the temporal patterns emerge: the baseline level remains relatively stable regardless of sampling date and the IR values are consistently above the baseline level.

**Figure 10:**
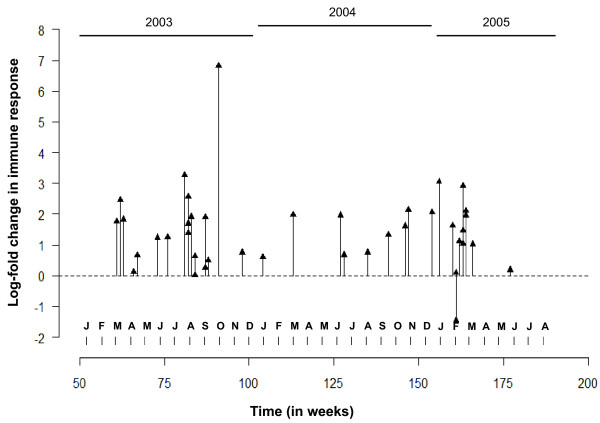
**Needle-plot of a time series of log-fold change in immune responses aligned accordingly to time of event, bi-monthly summary of cases, and average change in responses show the temporal clustering**.

A very peculiar feature emerges from Figure 9 (A and B), which suggests that the timing of diarrheal samples occurred in two temporal clusters approximately one year apart. To explore this phenomenon, we re-plot Figure 9 as a needle-plot (Figure 10). We label each month in the horizontal axes, express the change in IR as log-fold increase: Δ*Y_i _*= ln(Y_*t*2_/Y_*t*1_), and place individual values of log-fold increase at the time of each episode (these time points are marked by blue triangles in Figure 9A). Based on the density of events across time, we can depict two clusters of episodes: one between June-August, 2003 and the other - in February-March, 2005. This graphical depiction complements the findings presented in Figure 10, suggesting an increased probability of the seasonal exposure to *Cryptosporidium *during two time periods that are 3-6 months apart: from February to March and from June to September. The seasonal clustering can be further examined using regression models adapted to time series data [9]; however, such analysis typically requires a longer time period of observations and/or more events. When we extended this analysis of seasonality by considering the essential information related to residential locations (latitude and longitude) of children with diarrheal episodes and performed an analysis of spatio-temporal clustering, these two significant clusters of cryptosporidial diarrhea emerged [18].

**Figure 11:**
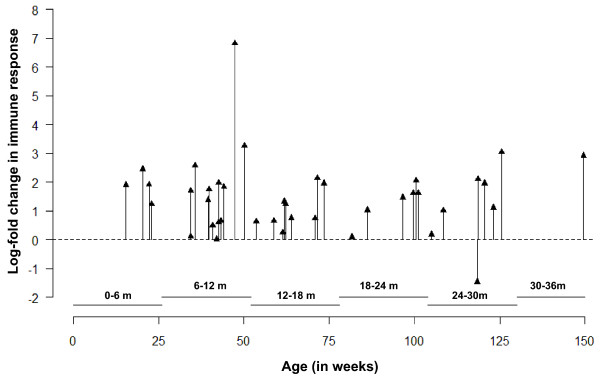
**Needle-plot of a time series of log-fold change in immune responses aligned accordingly to child's age at event of interest, summary of cases by age-groups, and average change in responses show age-related heterogeneity in immune response**.

While Figure 10 shows log-transformed ratios with respect to chronological time of a diarrheal episode for each child, similar analysis can be performed for understanding IR with respect to child age (see Figure 11). We divided the whole age range into seven equal age intervals of 6 months to closely mimic child development, which is important in acquiring infection. We then estimated the number of episodes for each interval (see Table 3), and found that the episodes are more likely to occur when children are between 6 to 18 month old, although the log-fold change in immune responses is relatively stable across all age categories (p-value = 0.525). This analysis complements our findings presented in Figure 8B and suggests an increased probability of developing the first infection due to *Cryptosporidium *between the ages of 6-18 months of age. Combined with the observations described above, we can assume that by the age of 24 months a child might be re-exposed multiple times.

**Table 3:** Summary statistics depicting the distribution of cases in different age groups and the change (log-fold) in their immune response (IR)

Age (in months)	No. of cases	Log-fold change in IR: Mean (SD)
0-6	4	1.9 (0.5)
6-12	13	1.8 (1.8)
12-18	9	1.1 (0.6)
18-24	6	1.3 (0.7)
24-30	7	1.1 (1.5)
30-36	1	2.9

## Conclusions

Human immunology is a growing field of research in which experimental, clinical, and analytical methods of many life science disciplines are utilized. Classic epidemiological study designs, including observational longitudinal birth cohort studies, offer strong potential for gaining new knowledge and insights into immune response to pathogens in humans. However, rigorous discussion of methodological issues related to designs and statistical analysis that are appropriate for longitudinal studies is lacking.

In this article, we addressed key questions of quality and validity of traditional and recently developed statistical tools applied to measures of immune responses and present examples that provide compelling evidence for the complexity of "field" sampling and the need for thorough examination of data originating from studies with a pre-post design. For this purpose we used data on humoral immune response associated with the first cryptosporidial diarrhea in a birth cohort of children residing in an urban slum in south India. The main objective of the statistical analysis was to detect the difference and derive inferences for a change in IR measured at two time points, before (pre) and after (post) an event of interest. We illustrated the use and interpretation of analytical and data visualization techniques including generalized linear and additive models, data-driven smoothing, and combinations of box-, scatter-, and needle-plots. We demonstrated that a detailed analysis allows us to describe new features of immune response to a pathogen in observational studies in humans. We borrowed our example from a birth cohort study, however the pre-post design is a widely used approach and the challenges and application of the presented methodology is not limited to the birth cohort setting. In our example, the event of interest was a diarrheal episode, but it could be any specific detectable episode, e.g. exposure, infection, symptom, sign, disease, vaccination, practically any procedure or condition that might affect immune response.

The obtained results have very important implications for future study designs and analyses, including considerations for scheduling post-event sampling. The demonstrated techniques can be adapted for longitudinal studies when multiple measurements are taken from study subjects. For instance, studies on immune response following vaccination (similar to [19,20]) will benefit from the proposed strategy of statistical analysis by accounting for the longitudinal nature of data collection.

We emphasized the need for analytical rigor in human immunology with respect to the use of statistical models as well as their tremendous capability to deal with issues related to the heterogeneity of immune response in humans and the complexity of research settings in "field" studies. Interplay between maternally acquired antibodies, prior exposure, time from exposure, and half-life of antibodies of different classes can all influence the development and duration of immune response in studies of vaccination and natural infection. We illustrated how the assumption of time irrelevance can be handled in a study with a pre-post design and how one can study the dynamics of IR in humans by considering the timing of response following an event of interest and seasonal fluctuation of exposure by proper alignment of time of measurements. This alignment of calendar time of measurements and a child's age at the event of interest can help to explore interactions between immune response, seasonal exposures and age at first infection, and ultimately the intrinsic features of infection dynamics.

We also emphasized the need for a better understanding of theoretical assumptions that form the foundation of proper application of statistical methods and the validity of such assumptions in the context of designed experimental settings. Even in a simple pre-experimental "one group pre-post design" with no additional samples for comparison it is extremely important to carefully examine underlying assumptions. The results of these studies can sufficiently inform researchers as to how to properly design the next steps and avoid costly mistakes. Furthermore, the intent of any life sciences study is to contribute to general knowledge so a thoughtful approach to each analytical step is necessary.

In human immunology (as in many other fields), it is widely accepted that experimental methods, e.g. randomized clinical trials, are the "gold standard" for causal inferences and measuring performance and the value of observational studies is belittled. While the shortcomings of non-experimental, or quasi-experimental, designs have been frequently discussed [21], a well conducted and thoughtfully evaluated pre-post study can provide highly valuable information, be rich in scope and can profoundly capture the process being investigated [22]. An effort should be made in order to supplement observational studies with powerful analytical tools similar to designed for clinical trials [23]. The complexity of multifaceted informational structures in data collected from large observational studies requires a thoughtful analytical plan, careful assessment of potential interactions, effect modifications, and confounding, selection of statistical methods and models appropriate to underlying assumptions, and an integrative interpretation of modeling results.

## List of Abbreviations

CI: confidence interval; IR: immune response measure; EU: ELISA (Enzyme Linked Immune Sorbent Assay) units; PCR-RFLP: polymerase chain reaction-restriction fragment length polymorphism; GLM: generalized linear model; GAM: generalized additive model; *t*-test: Student's *t*-test.

## Competing interests

The authors declare that they have no competing interests.

## Authors' contributions

RS prepared the first draft, designed the visual aids, assembled and analyzed the data; SRA performed immunological testing; HW and GK developed the immunological tests, designed and conducted the observational study; ENN conceived the process of statistical analyses and participated in developing visual aids and data analysis. All authors participated in manuscript preparation; all read and approved the final manuscript.

## Pre-publication history

The pre-publication history for this paper can be accessed here:

http://www.biomedcentral.com/1471-2288/12/1/prepub

## Supplementary Material

Additional file 1 **Longitudinal measures of immune responses of gp15 in 40 children**. Each child identified by unique number (**id**) provided three time points at specific dates (**date**) before, during and after a diarrheal episode, marked by categorical variable (**time**). For each child measures of immune responses of gp15 (**IR gp15**) are available for two time points: before and after an episode. Age in month with two decimals (**age in months**) is provided for each child at each time point. Click here for file
